# Childhood Adversity and Epigenetic Modulation of the Leukocyte Glucocorticoid Receptor: Preliminary Findings in Healthy Adults

**DOI:** 10.1371/journal.pone.0030148

**Published:** 2012-01-25

**Authors:** Audrey R. Tyrka, Lawrence H. Price, Carmen Marsit, Oakland C. Walters, Linda L. Carpenter

**Affiliations:** 1 Laboratory for Clinical Neuroscience, Mood Disorders Research Program, Butler Hospital, Providence, Rhode Island, United States of America; 2 Department of Psychiatry and Human Behavior, Brown Medical School, Providence, Rhode Island, United States of America; 3 Department of Pharmacology and Toxicology, Department of Community and Family Medicine, Dartmouth Medical School, Hanover, New Hampshire, United States of America; Wayne State University, United States of America

## Abstract

**Background:**

A history of early adverse experiences is an important risk factor for adult psychopathology. Changes in stress sensitivity and functioning of the hypothalamic-pituitary-adrenal (HPA) axis may underlie the association between stress and risk for psychiatric disorders. Preclinical work in rodents has linked low levels of maternal care to increased methylation of the promoter region of the glucocorticoid receptor (GR) gene, as well as to exaggerated hormonal and behavioral responses to stress. Recent studies have begun to examine whether early-life stress leads to epigenetic modifications of the GR gene in humans.

**Methods:**

We examined the degree of methylation of a region of the promoter of the human GR gene (*NR3C1*) in leukocyte DNA from 99 healthy adults. Participants reported on their childhood experiences of parental behavior, parental death or desertion, and childhood maltreatment. On a separate day, participants completed the dexamethasone/corticotropin-releasing hormone (Dex/CRH) test, a standardized neuroendocrine challenge test.

**Results:**

Disruption or lack of adequate nurturing, as measured by parental loss, childhood maltreatment, and parental care, was associated with increased *NR3C1* promoter methylation (p<.05). In addition, *NR3C1* promoter methylation was linked to attenuated cortisol responses to the Dex/CRH test (p<.05).

**Conclusions:**

These findings suggest that childhood maltreatment or adversity may lead to epigenetic modifications of the human GR gene. Alterations in methylation of this gene could underlie the associations between childhood adversity, alterations in stress reactivity, and risk for psychopathology.

## Introduction

Many decades of research in rodents, non-human primates, and humans have documented the impact of early experiences on the neurobiological mechanisms regulating stress responses and mood and anxiety disorders. Young children are dependent on caregivers for their basic physical, social and emotional needs, and in addition undergo substantial developmental changes in neural pathways involved in regulating emotion and behavior. As a result, disruption of early care-giving can produce profound and long-lasting changes in these neurobiological and behavioral systems [1]–[3]. Early-life stress is a risk factor for major depression, post-traumatic stress disorder, and drug abuse, among other conditions [4], [5].

Alteration of basal and stress-induced activity of the hypothalamic-pituitary-adrenal (HPA) axis is implicated in the pathogenesis of these disorders. Chronic alterations of HPA axis activity have been shown in rodents and non-human primates exposed to disruptions of parental care such as maternal separation [6], [7] and maternal neglect [8], and in humans with childhood parental loss, and neglect or other forms of childhood maltreatment [2], [9]–[17]. Elevated glucocorticoids impair neuronal growth and survival [18], diminish neurotrophins and modify immune function [19], and accelerate cellular aging [19], [20], all of which have been associated with both early-life stress [18], [21]–[23] and major depression [18], [24]–[26].

Preclinical work implicates epigenetic changes to the gene for the type II glucocorticoid receptor (*NR3C1*) as a mechanism underlying the neuroendocrine effects of environmental adversity. Epigenetic alterations to DNA influence gene expression but do not change the nucleotide sequence of DNA. Methylation is a stable form of epigenetic modification which alters gene expression via effects on transcription factor binding [27]. Methyl residues chemically modify regions of DNA where a cytosine nucleotide occurs next to a guanine nucleotide via a phosphodiester bond (CpG sites). As reviewed by Weaver [27] and Champagne and Curley [28], low levels of maternal care in rodents result in greater methylation of the promoter region of the hippocampal glucocorticoid receptor (GR) gene (exon 1_7_ of the *NR3C1* promoter), which interferes with binding of nerve growth factor inducible protein A (NGFI-A), a transcription factor. Greater methylation reduces *NR3C1* gene expression, which results in decreased numbers of GRs in the hippocampus and exaggerated hormonal and behavioral sequelae of stress.

Two published investigations have examined associations of early experiences with epigenetic modification of the promoter of the human GR gene *NR3C1*. Both studies focused on the exon 1F region of the promoter, which is homologous to the rat exon 1_7_ and contains the NGFI-A binding site. Oberlander and colleagues [29] examined mixed mononuclear cells from cord blood of 82 infants and found that increased maternal depressed mood in the third trimester was linked to increased methylation at CpG 1 and 2 in this region, as well as CpG 3, an NGFI-A binding site. CpG 3 methylation was also positively associated with salivary cortisol responses to infant stimulation at three months.

The second human study on this topic examined DNA from postmortem hippocampal tissue from 36 men [30]. Proxy-based retrospective interviews identified 12 suicide victims with a history of childhood abuse, 12 suicide victims without abuse, and 12 control subjects without known abuse who died from another cause. The suicide/abuse victims had increased cytosine methylation of the *NR3C1* promoter and decreased levels of GR mRNA compared to the other groups. Patch-methylated *NR3C1* promoter constructs that mimicked enhanced DNA methylation showed a corresponding decrease in binding of NGFI-A and NGFI-A–inducible gene transcription. These findings suggest that childhood abuse may result in decreased transcription of hippocampal GR in humans.

Based on the compelling body of preclinical work, the two previous studies in humans, and our prior findings of enduring neuroendocrine alterations in adults with a history of childhood adversity, we conducted the present study to test the hypothesis that increased leukocyte *NR3C1* promoter methylation is associated with adverse childhood experiences and with attenuated cortisol responses to a standardized neuroendocrine challenge test. We report here that childhood adversity is associated with greater *NR3C1* methylation, and that individuals with greater levels of *NR3C1* methylation have attenuated cortisol responses to the dexamethasone (Dex)/corticotropin-releasing hormone (CRH) test.

## Materials and Methods

### Ethics Statement

This study was approved by the Butler Hospital Institutional Review Board, and after complete description of the study to the subjects, written informed consent was obtained.

### Subjects

Ninety-nine participants, 58 women and 41 men, aged 18–59 (27.3±10.4) years, were recruited for several related studies of stress and HPA axis function using local, newspaper, and internet advertisements directed toward healthy adults, individuals with a history of early parental loss, and adults with a history of early-life stress. Subjects were compensated $175 for their time and effort spent participating in the study.

Following a telephone screening to determine preliminary eligibility, participants completed a medical history, physical examination, neurological examination, electrocardiogram, and standard laboratory studies to rule out pregnancy or major medical illness, including, but not limited to, endocrine disease, allergy symptoms, or a history of brain injury or seizures. Also excluded were individuals with use of psychotropics, beta blockers, angiotensin-converting enzyme inhibitors, ketoconazole, metyrapone, or corticosteroids. Oral contraceptives were allowed, with usage accounted for in analyses of cortisol concentrations.

### Demographic Characteristics

In addition to age and sex, we measured weight and height and calculated body mass index [BMI, weight (Kg)/height (M)^2^]. Because we were interested in the socioeconomic conditions of the adult participants during their childhood, we used the following statements to determine socioeconomic adversity: 1) I grew up in an area of high crime; 2) My family was generally financially stable when I was growing up, and all of my basic needs (food, shelter, and clothing) were met during my childhood. Sixteen subjects were considered to have socioeconomic adversity during childhood based on adverse scores on either of these statements.

### Behavioral and Stress Measures

#### Psychiatric Symptoms and Diagnoses

Axis I psychiatric diagnoses were assessed using the Structured Clinical Interview for DSM-IV [SCID; [31], and participants were excluded if they met criteria for any current or past disorder except simple phobia in order to isolate effects of early adversity. The Inventory for Depressive Symptoms, Self Report [32], the State-Trait Anxiety Inventory [33], and the Perceived Stress Scale [34] were included to assess subclinical symptoms and recent perceived stress.

#### Parental Bonding Index: Parental Care [35]

The Parental Bonding Index assesses parenting as perceived by the child and assessed retrospectively in adulthood. A higher score on the “Care” factor reflects parental warmth, affection, and involvement, while a lower score reflects greater parental coldness, rejection, and detachment. This measure has high stability and validity over 20 years of follow-up and is not substantially influenced by depressed mood or neuroticism [35], [36]. The mean of the maternal and paternal scales was used for assessment of Parental Care.

#### Childhood Parental Loss

Loss of a parent before age 18 (N = 22) included death (N = 12) and/or prolonged separation/desertion (i.e., parent deserted for at least six months with no attempts at contact or responses to child's attempts (N = 14)).

#### The Childhood Trauma Questionnaire, 28-item version [37]

This measure generates a total score summarizing five types of childhood maltreatment (physical, sexual, and emotional abuse, physical and emotional neglect), with high internal consistency, test-retest reliability, and convergent validity [38].

### NR3C1 Promoter Methylation Sequencing

**Figure 1:**
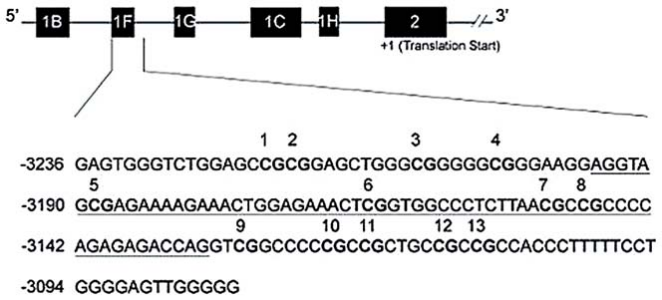
Schematic representation of human *NR3C1* 5′ regulatory region, and the specific region analyzed by bisulfite pyrosequencing. The upper diagram represents the multiple first exons, and the second exon (2) which represents the translational start. All numbering is based on the translational start site (+1), which is located 13 nucleotides within the start of exon 2. The lower region is the region analyzed by pyrosequencing, with the 13 CpG sites in bold and numbered above the sequence, and the full exon 1F region underlined.

DNA was extracted using standard methods from whole blood. Blood samples were thawed only once for DNA extraction, and modified DNA was thawed only once prior to sequencing. Sodium bisulfite modification of DNA was performed using the EZ DNA Methylation Kit (Zymo Research, Orange, CA) following the manufacturer's protocol. Methylation at the *NR3C1* promoter region was examined with a quantitative pyrosequencing approach following the method of Oberlander and colleagues [27]. The region analyzed contains 13 CpGs and encompasses exon 1F, the human homologue of the rat exon 1_7_ (Figure 1). Sodium bisulfite-modified, fully-methylated referent positive control and fully-unmethylated (whole genome amplified) negative control DNA (Qiagen, Valencia CA) was examined with each batch. Peripheral blood lymphocyte DNA that was not sodium bisulfite-modified served as a control for non-specific amplification. Methylation quantification was performed using the Pyromark Software (Qiagen). The percent of alleles that were methylated in the cell population examined was used in statistical analyses.

### Dex/CRH Test

On a subsequent visit, 88 participants completed the Dex/CRH test. The night before the test, an oral dose of dexamethasone 1.5 mg was self-administered at 11 p.m. The following day, subjects arrived at 12:00 p.m. and were given lunch. A topical anesthetic (lidocaine 2.5% and prilocaine 2.5%) was applied to the subject's forearm between 12:30 and 12:45 p.m. At 1:00 p.m. an indwelling intravenous (IV) catheter was inserted in the forearm by a highly experienced research nurse. Subjects then remained in a semi-recumbent position throughout the procedure except to use the bathroom. They were permitted to read or watch pre-selected films that did not contain emotionally-charged material. Vital signs were monitored throughout the test. At 3:00 p.m., CRH 100 µg (corticorelin ovine triflutate, Acthrel, Ferring Pharmaceuticals, Inc.) reconstituted in 2 ml of 0.9% NaCl solution was infused intravenously over 30 seconds. Blood samples were drawn at 2:59 p.m., 3:30 p.m., 3:45 p.m., 4:00 p.m., 4:15 p.m., and 5:00 p.m. and assayed for cortisol.

### Hormone Assays

Plasma cortisol concentrations were assayed in duplicate using the Gamma Coat cortisol I-125 coated-tube radioimmunoassay kit (INCSTAR Corp., Stillwater, Minnesota). Intra-assay and interassay CVs observed for quality assessment samples (5 and 20 µg/dL) were less than 5% and 10%, respectively. Area under the curve over time for cortisol was calculated and log transformed due to positive skew.

### Statistical Analysis

To minimize the number of comparisons and mitigate the possibility of type I statistical errors, we selected a small number of relatively distinct domains of childhood adversity and CpG sites for *a priori* hypotheses regarding the effect of adversity on CpG methylation. Childhood adversity measures included Maltreatment (Childhood Trauma Questionnaire total score), Parental Care (subscale of the Parental Bonding Instrument), and Parental Loss (dichotomous). As expected, these measures were moderately inter-correlated (correlations with other adversity measures for Parental Loss were r = .27 and .30, Parental Care r = .27 and .65, and Maltreatment r = .30 and .65). Although the Maltreatment and Parental Care domain scales showed substantial intercorrelations, the magnitude of the correlations revealed substantial unique variance, indicating that the three childhood adversity measures were not redundant. To test for combined effects of these variables, an Adversity Index was composed of the three childhood adversity variables, with one point each for Parental Loss, Maltreatment (upper tertile of the distribution), and Low Parental Care (lower tertile of the distribution). The distribution of scores on this sum scale for number of adversity experiences was as follows: 0, N = 52; 1, N = 17; 2, N = 20; and 3, N = 10.

We selected CpG sites 1–3 in this region based on the findings of Oberlander and colleagues [27], and we also included CpG 4, because NGFI-A binds at CpG 3 and 4. Intercorrelations of methylation at CpG sites 1–4 were relatively small (r = .05–.38), indicating that these sites are regulated in a largely independent fashion. Following Oberlander and colleagues, we examined additional CpG sites in this region (CpG 5–13) in an exploratory fashion, and we regard any findings as hypothesis-generating. CpG sites 5 and 6 had small associations with all other CpG sites (range r = −.09–.33). CpG sites 7–13 showed small associations with CpG sites 1–6 (range r = −.04–.33), but were very highly intercorrelated (r = .32–.85; each CpG site had at least one correlation with another of these sites that was greater than 0.5, and several were in the .7 and .8 range). Therefore, we calculated the mean percent methylation for CpG 7–13 and used this single variable in the exploratory analyses.

Outliers, defined as values more than three standard deviations from the mean were Winsorized by setting them to the next highest value within three standard deviations. There were three outliers for CpG 2, two outliers for CpG 3, and one outlier for CpG 4. Of the exploratory sites, there was one outlier for CpG 6 and two outliers for the mean of CpG 7–13. Demographic characteristics, as well as recent perceived stress levels and subsyndromal symptoms of depression and anxiety, were examined and controlled when statistically significant. Partial correlations were used to test for associations of the continuous adversity variables and the Adversity Index. For the dichotomous Parental Loss variable, analysis of covariance was used to test for effects, but partial correlations were displayed in the table for consistency.

## Results

### Potential Covariates

Age was correlated with greater methylation at several CpG sites. Age was also associated with scores on the Parental Care scale and Childhood Trauma Questionnaire, so we controlled for age in the analyses. Age, sex, and estrogen use, known determinants of cortisol response, were controlled in analyses of cortisol responses. BMI, estrogen use, and childhood socioeconomic adversity were not associated with cytosine methylation in this region, and were not considered further in the analyses.

### Childhood Adversity and Percent Methylation at Hypothesized CpG Sites

**Figure 2:**
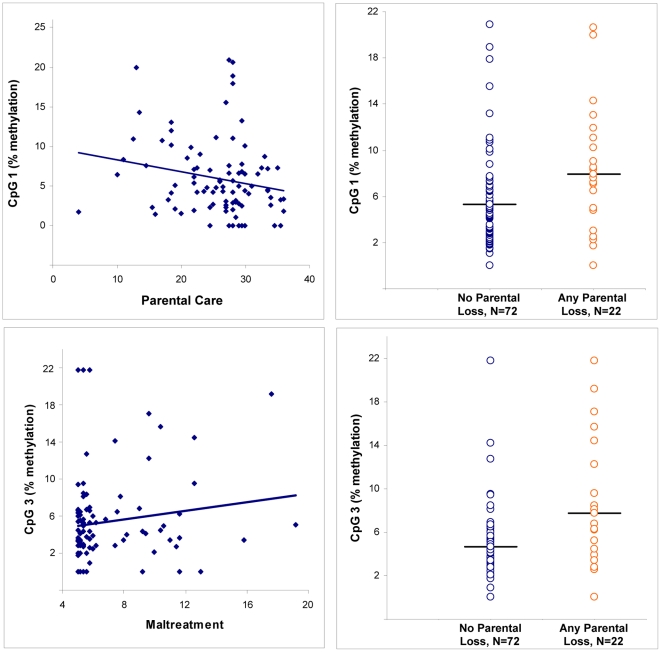
Significant Associations of Childhood Adversity Variables and Percent Methylation at Predicted CpG Sites (1–4). The Parental Care scale of the Parental Bonding Instrument reflects parental warmth, affection, and involvement; lower scores reflect greater parental coldness, rejection, and detachment. Maltreatment reflects the total score of Childhood Trauma Questionnaire. Parental loss is defined as loss of a parent before age 18, including death (N = 12) and/or prolonged separation/desertion (N = 14).

The partial correlations and analyses of covariance, adjusting for age and testing effects of childhood adversity variables at CpG sites 1–4, are shown in Table 1 and depicted in Figure 2. Greater methylation at CpG 1 was associated with lower levels of Parental Care and with Parental Loss (r = −.23, p = .03 and F = 5.9, p = .02, respectively). Methylation at CpG 2 was not associated with any of the childhood adversity variables. Greater methylation at CpG 3 was linked to Maltreatment (r = .23, p = .03), as well as to Parental Loss (F = 8.2, p = .005). Methylation at CpG 4 was not significantly associated with any of the adversity variables. Similarly, the Adversity Index was positively correlated with CpG 1 (r = .22, p = .03) and CpG 3 (r = .22, p = .04), but not CpG 2 or 4.

**Table 1:** Associations of Childhood Adversity Measures and Percent Methylation at Hypothesized CpG Sites (1–4).

	CpG 1	CpG 2	CpG 3	CpG 4
Maltreatment	**.173**	.103	**.228**	.171
	**.097**	.324	**.028**	.102
Parental Care	**−.234**	.069	−.084	−.094
	**.026**	.516	.429	.377
Parental Loss	**.228**	.057	**.257**	.059
	**.030**	.593	**.014**	.576

Note: Statistic represents partial correlation coefficients (r) controlling for age. P values are shown underneath the coefficients. Significant and trend-level effects are highlighted in bold.

### Childhood Adversity and Percent Methylation at Exploratory CpG Sites

Exploratory analyses of methylation at CpG sites 5, 6 and the mean of CpG 7–13 did not reveal any effects of childhood maltreatment, quality of parenting, or loss of parent, although there was a trend for low Parental Care to be associated with greater methylation at CpG 5 (p = .065). The Adversity Index was not associated with these exploratory sites.

### Dex/CRH Test and Methylation at Hypothesized and Exploratory CpG Sites

**Figure 3:**
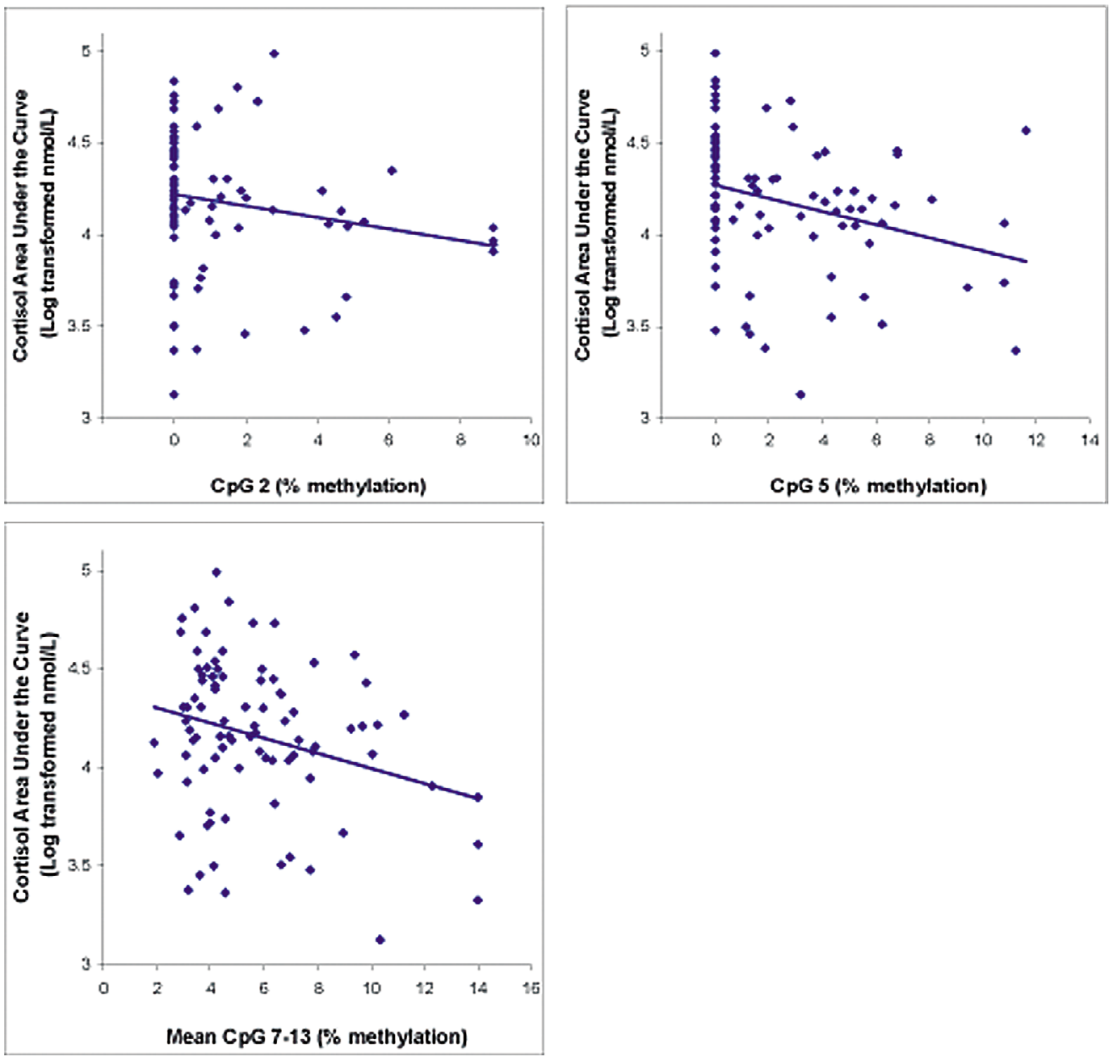
Significant Associations of Cortisol AUC in the Dex/CRH Test and Percent Methylation at CpG Sites. AUC is defined as Area Under the Curve.

Partial correlations, controlling for age, sex, and estrogen use, revealed a negative association of cortisol area under the curve with percent methylation at CpG 2 (r = −.23, p = .03, Figure 3). Methylation at CpG sites 1, 3, and 4 were not associated with cortisol response to the Dex/CRH test. The exploratory CpG sites 5 and the mean of 7–13 both showed negative associations with cortisol response (r = −.25, p = .03 and r = −.23, p = .04, respectively; Figure 3).

### Subsyndromal Symptoms and Current Stress

Partial correlations, controlling for age, revealed that subsyndromal symptoms of depression and anxiety, as indexed by the Inventory for Depressive Symptoms Self Report and the State-Trait Anxiety Inventory, were not significantly associated with methylation at any of the four predicted CpG sites. There was a trend-level association of increased methylation at CpG4 with depressive symptoms (p = .056), but controlling for depressive symptoms did not alter any of the childhood adversity or cortisol findings. Perceived stress showed a significant positive association with methylation at one site, CpG 2 (r = .23, p = .03), but controlling for this variable did not alter the adversity findings or the cortisol findings.

For the exploratory CpG sites, neither state-trait anxiety nor recent perceived stress was associated with methylation. Subsyndromal depressive symptoms showed a significant negative association with methylation at CpG 6 only (r = −.20, p = .049), but controlling for this variable did not substantively change any of the adversity or cortisol results at the exploratory CpG sites.

## Discussion

We found that healthy adults reporting a history of childhood adversity (loss of a parent, maltreatment, poor quality parenting) had increased cytosine methylation of the promoter region of the GR gene *NR3C1*. In addition, methylation of this region was associated with attenuated cortisol responses to the Dex/CRH test in adulthood. This is the first study to demonstrate that variation in human parenting experiences is linked to epigenetic modulation of the leukocyte GR gene. Our results are consistent with findings of the two prior human studies (using hippocampal tissue and infant cord blood, respectively) of epigenetic modulation of this gene in association with childhood maltreatment [30] and prenatal exposure to maternal depressed mood [29]. In rodents, low levels of maternal care during early postnatal life are associated with increased cytosine methylation and decreased expression of the hippocampal GR (*NR3C1*) promoter [27]. Given previous findings on childhood maltreatment and loss on risk for psychopathology, the effect of promoter methylation on GR expression and glucocorticoid responses, and the role of HPA axis dysfunction in depressive and anxiety disorders, the present findings suggest that epigenetic changes to this gene could be a key mechanism for effects of childhood adversity on risk for these disorders.

The transcription factor NGFI-A binds at CpG 3 and 4, and we found that CpG 3 methylation was associated with loss of a parent and maltreatment in childhood. Methylation at CpG 4 was not significantly linked to these adversity measures. Our findings extend the observations of Oberlander and colleagues [27], who found that cytosine methylation at CpG 3 was associated with exposure to maternal depression in the third trimester, and with increased infant cortisol responses. Methylation at this site may alter transcription; methylation at the NGFI-A binding site in hippocampus has been shown to reduce GR expression in rodents [39]–[41] and humans [27], [30]. However, this site was not methylated in mice treated with oral corticosterone [42] or rat pups subjected to maternal separation [43]. In humans, one study found that the NGFI-A binding site was unmethylated in post mortem hippocampal tissue from 32 individuals, most with Parkinson's or Alzheimer's disease, but no information on early environmental stressors was available in this study [44]. A recent examination of post-mortem brain from donors with major depression (but without documented child abuse) found reduced expression of hippocampal *NR3C1* exon 1F [45]. However, promoter 1F was uniformly unmethylated and instead, NGFI-A was downregulated. This suggests that while altered GR transcription may be common to childhood abuse and major depression without early adversity, the mechanism may differ in the two conditions.

Our findings of greater methylation at CpG 1 in association with low Parental Care and with loss of a parent in childhood also extend the findings of Oberlander and colleagues [27] of increased infant methylation of CpG 1 in association with maternal depressed mood in the third trimester. The additive Adversity Index was also positively correlated with both CpG 1 and 3, indicating that a greater number of different adverse childhood experiences is associated with more methylation at NR3C1. Our findings appear to be specific to early environment stress and are not accounted for by current psychopathology or subsyndromal symptoms.

None of our adversity measures was significantly associated with methylation at CpG 2 or the exploratory CpG sites (although there was a trend for low Parental Care to be associated with greater methylation at CpG 5). Cortisol response to the Dex/CRH test showed negative associations with CpG site 2 and the exploratory sites CpG 5 and the average of sites 7–13. That we found *attenuated* cortisol responses contrasts with findings of *enhanced* cortisol responses in rodents with increased *NR3C1* promoter methylation and decreased receptor expression [27], as well as with increased cortisol responses in infants with greater cytosine methylation at CpG 3 [29]. It therefore seems unlikely that our cortisol findings are a direct effect of methylation-induced decreases in GR expression. Our results are consistent, however, with prior reports of blunted cortisol responses in association with early-life stress from our group [12]–[14], [46] and others (e.g., [16], [47]). The mechanism of this effect is unknown, but may involve a trajectory of initial hyper-activation of the HPA system in response to excessive and prolonged stress exposure progressing to a counter-regulatory adaptation state of chronic adrenal stress hyporeactivity [6], [48], [49]. This may be influenced by the developmental timing or chronicity of early-life stress; unfortunately we do not have these measures in the present study. We do not have gene expression data in this sample, and our measure of HPA axis function was limited to the Dex/CRH test. This test is a sensitive measure of HPA axis dysfunction, but because it combines a large dose of dexamethasone with corticotropin-releasing hormone administration, it does not permit assessment of feedback sensitivity or other specific components of the axis that may explain the findings. Possible mechanisms include compensatory down-regulation of corticotropin-releasing hormone or adrenocorticotropic hormone receptors, or increases in GR negative feedback sensitivity, in response to or in addition to changes in GR expression.

Exposure to stress and trauma are increasingly thought to underlie the neuroendocrine abnormalities seen in some patients with depressive and anxiety disorders. Abnormal HPA axis activity may play a central role in the pathogenesis of depressive and anxiety disorders, perhaps in those with excessive stress exposure. Preclinical studies show that chronic stress and glucocorticoid administration result in hippocampal atrophy [50], which has also been documented in adults with a history of childhood maltreatment, major depression, or post-traumatic stress disorder [50], [51]. In contrast, stress and glucocorticoids can produce excitability and dendritic remodeling in the amygdala, which mediates anxiety and fear responding [52]. Thus, alterations in HPA axis activation in response to early deprivation may alter the structure and activity of brain regions involved in mood and anxiety disorders. We excluded individuals with current or past Axis I psychiatric disorders in the current study. This allowed us to isolate the effects of early adversity, but it is important to note that this exclusion likely resulted in a sample of individuals who are especially resilient to psychopathology, in addition to those who are at risk but have not yet developed a disorder.

In this study we examined the leukocyte GR gene, whereas the previous human investigations of the effect of exposures examined cord blood or hippocampal tissue. The similarity of our findings to those of the prior human studies, as well as animal work on the hippocampal GR in relation to variation in maternal care, is encouraging, but data regarding the differential control of expression of the GR in various tissues are very limited. Some evidence indicates similarities in the regulation of this gene in brain and peripheral tissues. Lee and colleagues [42] found that chronic oral corticosterone administration caused anxiety-like behavior and a decrease in hippocampal and blood mRNA levels of *NR3C1*. Moreover, the relevance of regulation of leukocyte glucocorticoid receptor expression for stress-related disorders is supported by evidence of abnormal sensitivity and expression of this receptor in leukocytes of patients with major depression and post-traumatic stress disorder [53], [54].

Our findings of age-related alterations to the extent of methylation are consistent with other reports that identify gene-specific associations between methylation and aging [55], [56]. In addition, in this sample, older subjects reported higher levels of Maltreatment and lower Parental Care than younger participants (and age was controlled in the analyses). This may be a cohort effect, but it is possible that it is due to an age-related reporting or memory bias. We studied several related childhood experiences that commonly co-occur and may have similar or redundant epigenetic effects. It is notable that the Adversity Index, the sum of the various childhood adversities, was positively associated with the degree of *NR3C1* methylation. Childhood parental loss, which represents a more objective event than some other types of experiences and is less likely to co-occur with maltreatment than other forms of adversity, was the strongest predictor of CpG methylation (and was not associated with age). It should be noted that the effects of childhood parental loss may be mitigated by personal and familial characteristics that influence adaptation to loss (e.g., [14], [57]).

Because we studied a mixed population of leukocytes, our findings could be influenced by the relative composition of these cells, which can be altered during infection and other conditions. Glucocorticoids have strong anti-inflammatory effects, and glucocorticoid receptors are present in several leukocyte cell types, including lymphocytes, neutrophils, eosinophils, and macrophages [58]. However, it is unlikely that inflammatory conditions explain our findings because 1) we excluded subjects with acute or chronic illness, allergy symptoms, abnormal blood counts, or the use of antibiotics, antihistamines, or corticosteroids, and 2) any such confounding effect would have to occur differentially in those with adversity.

In summary, we found that early-life stress, in the form of loss of a parent during childhood, maltreatment, and low parental care, was associated with epigenetic changes to the promoter region of the glucocorticoid receptor gene. In addition, methylation of the promoter region of this gene was linked to alterations in HPA axis function. These findings, together with previous research in rodents and two prior studies in humans, provide preliminary support for the hypothesis that altered expression of the glucocorticoid receptor due to cytosine methylation of the gene promoter could be a mechanism of the neuroendocrine effects of early-life stress, and could predispose to the development of major depression and post-traumatic stress disorder. However, our effect sizes tended to be modest in magnitude, different CpG sites were associated with the associations of childhood adversity measures and the cortisol response to the Dex/CRH test, and we did not have gene expression data available. This study will need to be replicated in order to draw firm conclusions about the findings. Further work is also needed to determine whether these findings are specific to lymphocytes and whether this reflects changes in central regulation of the glucocorticoid receptor in brain regions involved in stress responses and mood and anxiety disorders.
